# Immune checkpoint blockade targets macrophage PD-1 to exacerbate metabolic dysfunction

**DOI:** 10.1097/IN9.0000000000000079

**Published:** 2026-04-30

**Authors:** Zikuan Song, Christian Frezza

**Affiliations:** 1 Faculty of Medicine, Institute for Metabolomics in Ageing, Cluster of Excellence Cellular Stress Responses in Aging-associated Diseases (CECAD), University Hospital Cologne, University of Cologne, Cologne, Germany; 2 Faculty of Mathematics and Natural Sciences, Institute of Genetics, Cluster of Excellence Cellular Stress Responses in Aging-associated Diseases (CECAD), University of Cologne, Cologne, Germany; 3 Center for Molecular Medicine (CMMC), University of Cologne, Cologne, Germany

**Keywords:** cancer, metabolism, immunity, programmed cell death protein 1, macrophages

## Abstract

Immune checkpoint inhibitor therapies induce metabolic dysfunction. A study by Wu et al now pinpoints macrophage programmed cell death protein 1 (PD-1) as a key molecular mediator of the anti-PD-1 treatment-triggered exacerbation of systemic metabolic disorders. Macrophage PD-1 blockade disrupts the moonlighting function of PD-1 in suppressing endoplasmic reticulum stress-mediated inflammatory responses, thereby impairing adipose tissue thermogenesis, reducing energy expenditure, and ultimately leading to systemic metabolic dysfunction.

Immune checkpoint inhibitors (ICIs) have revolutionized cancer therapy by harnessing the power of patients’ own immune system to combat tumors ^[1,2]^. Within this therapeutic framework, the programmed cell death protein 1 (PD-1) and programmed death-ligand 1 (PD-L1) pathway has been a central focus ^[3]^. The interaction between PD-1 and PD-L1 serves as a potent inhibitory “brake” on the immune system and is commonly exploited by cancer cells to evade immune destruction ^[4]^. To block this interaction and restore anti-tumor immunity, therapeutic antibodies targeting PD-1 or PD-L1 have been widely used in clinical practice over the past decade and have achieved promising efficacy in tumor control across multiple cancer types ^[5]^. Despite these successes, increasing evidence has shown that disrupting the immune checkpoint pathways induces a spectrum of immune-related adverse events ^[6]^. Among these, an increased risk of metabolic dysfunctions upon ICI treatments, such as hyperglycemia and diabetes, has been gradually appreciated ^[7]^. However, the biological mechanisms underlying this ICI therapy-induced disturbance of metabolic homeostasis remain elusive. This major knowledge gap considerably hinders the therapeutic benefits and long-term safety of ICI therapies.

A recent work published in *Cell Metabolism* in January 2026 by Wu et al set out to address this question ^[8]^. By injecting mice with an anti-PD-1 antibody, the authors demonstrated that PD-1 blockade significantly exacerbated high-fat diet (HFD)-induced obesity and systemic metabolic dysfunction by reducing energy expenditure without affecting food intake. Importantly, these observed metabolic abnormalities, such as hyperglycemia, glucose intolerance, and insulin resistance, align with clinical observations of ICI-associated adverse events ^[9]^. To identify the specific immune cell population through which anti-PD-1 antibody mediates these effects, Wu et al generated myeloid-, neutrophil-, and T-cell-specific PD-1 knockout (KO) mice. Notably, only myeloid-specific PD-1 deletion fully phenocopied the metabolic dysfunctions triggered by systemic PD-1 blockade. These data, for the first time, reveal an unexpected role for myeloid PD-1 as a key nexus linking anti-PD-1 therapies to their associated metabolic adverse events.

Mechanistically, myeloid PD-1 deficiency triggered systemic inflammation and accumulation of inflammatory macrophages in adipose tissues, concomitant with impaired white adipose tissue (WAT) thermogenesis and browning (Figure 1). It is perhaps not surprising that the removal of a classical “immune brake” signaling pathway in myeloid cells results in a pronounced inflammatory phenotype. Nevertheless, one of the most intriguing and novel findings of this study is the identification of a previously unknown function of macrophage PD-1 in regulating cellular endoplasmic reticulum (ER) stress and ultimately HFD-induced metabolic disorders. Specifically, the authors showed that liposaccharide, a well-established tool for mimicking obesity-associated chronic inflammation, triggered the phosphorylation of Unc-51-like autophagy activating kinase 1 (ULK1) in a mammalian target of rapamycin (mTOR)-dependent manner in macrophages. They also found that upon activation, ULK1 phosphorylates PD-1 at threonine 250 (T250), stabilizing PD-1 by preventing its ubiquitination and proteasomal degradation mediated by the E3 ligase F-box protein 38 (FBXO38). Subsequently, macrophage PD-1 T250 phosphorylation promoted its interaction with inositol-requiring enzyme 1α (IRE1α) and enabled its moonlighting function in inhibiting IRE1α phosphorylation and dimerization, thereby suppressing ER-stress-mediated inflammatory responses, particularly pro-inflammatory cytokine productions. Importantly, both pharmacological suppression and genetic deletion of IRE1α completely rescued HFD-induced aggravation of metabolic dysfunctions in myeloid-specific PD-1 KO mice. Collectively, these data underscore the essential role of myeloid mTOR–ULK1–PD-1-IRE1α axis in coordinating the interplay between ICI therapy and metabolic dysfunctions. This newly uncovered non-canonical function of myeloid PD-1 provides compelling rationale that may prove helpful in combating not only ICI-triggered metabolic disorders but also a diverse array of obesity-related metabolic diseases.

**Figure 1.**
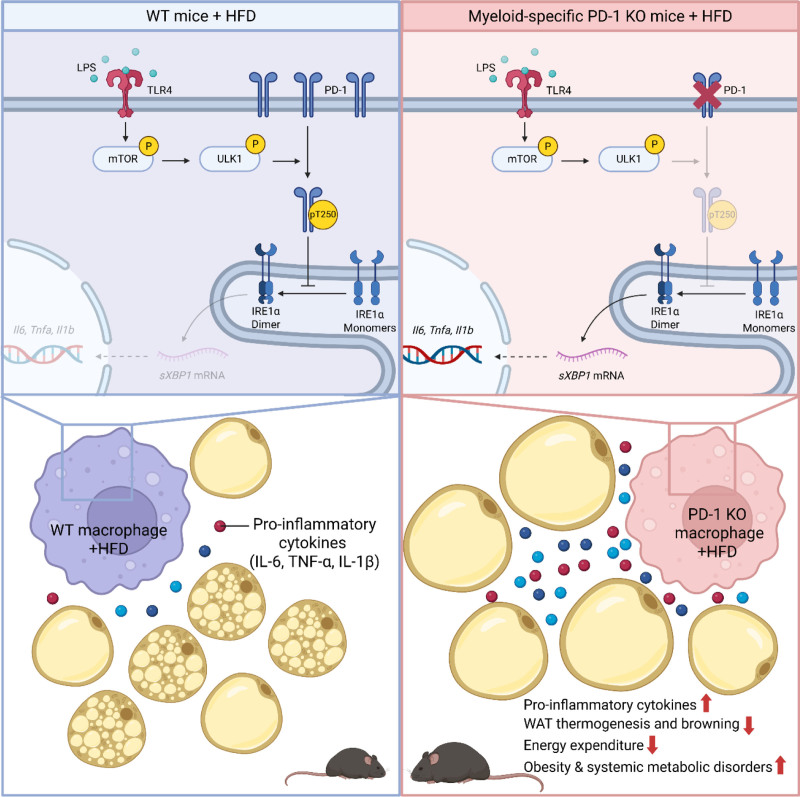
**Macrophage PD-1 deficiency exacerbates high-fat diet (HFD)-induced obesity and metabolic disorders.** In macrophages, lipopolysaccharide (LPS) activates the phosphorylation of Unc-51-like autophagy activating kinase 1 (ULK1) in a mammalian target of rapamycin (mTOR)-dependent manner. Under wildtype (WT) conditions (on the left), activated ULK1 phosphorylates PD-1 at threonine 250 (T250) and stabilizes PD-1. After that, macrophage PD-1 T250 phosphorylation promotes its interaction with inositol-requiring enzyme 1α (IRE1α) and thereby blocks IRE1α dimerization and suppresses subsequent endoplasmic reticulum (ER) stress. In contrast, in PD-1 knockout (KO) macrophages (on the right), due to the absence of PD-1 and its T250 phosphorylation, the blockade of IRE1α dimerization and activation is lifted. Consequently, dimerized IRE1α triggers ER-stress-mediated inflammatory responses via *Xbp1* (X-box binding protein 1) mRNA splicing and pro-inflammatory gene expression increases, including interleukin-6 (*Il6*), tumor necrosis factor-α (*Tnfa*), and Interleukin-1β (*Il1b*). These elevated pro-inflammatory cytokine levels impair white adipose tissue (WAT) thermogenesis and browning and decrease overall energy expenditure, thereby exacerbating HFD-induced obesity and systemic metabolic dysfunction. Figure is created in BioRender.com. PD-1, programmed cell death protein 1.

While this study elucidates detailed and convincing mechanisms that position macrophage PD-1 at the center of ICI-induced disruption of metabolic homeostasis, several important questions about the described molecular mechanisms and their clinical applicability remain.

At the molecular level, the authors demonstrated that phosphorylation of PD-1 T250 suppresses IRE1α activation, thereby attenuating ER stress and pro-inflammatory cytokine production in macrophages. However, the downstream mechanisms by which these pro-inflammatory cytokines impair WAT thermogenesis and browning remain unclear. Specifically, whether pro-inflammatory cytokines directly inhibit uncoupling protein 1 expression in adipocytes or act indirectly through sympathetic signaling requires clarification. In addition, whether other markers of adipogenesis and thermogenesis are changed in WAT under these conditions warrants further investigation to comprehensively understand the impact on WAT physiology. Moreover, while myeloid-, neutrophil-, and T-cell-specific PD-1 KO mice were individually used to identify the essential role of myeloid PD-1 in mediating metabolic disorders, the mechanism of action under anti-PD-1 antibody treatment warrants further clarification. Specifically, it remains unclear whether the metabolic phenotypes observed following PD-1 blockade are only modulated by directly targeting M1/M2 macrophages or also arise from indirect effects mediated through T-cell-dependent immune regulation.

This study also provides extensive and compelling data suggesting that anti-PD-1 antibody treatment perturbs the macrophage mTOR–ULK1–PD-1-IRE1α axis, leading to systemic inflammatory responses. Yet, the specific step in the mechanistic cascade that is directly disrupted by anti-PD-1 treatment remains unclear. In particular, future investigations are required to determine whether anti-PD-1 antibodies generally promote PD-1 degradation or, more specifically, block PD-1 T250 phosphorylation, interfere with PD-1 intracellular trafficking, or hinder PD-1–IRE1α interaction. Clarifying these mechanisms would be essential for developing therapeutic strategies to alleviate ICI-induced metabolic dysfunctions without compromising its anti-tumor efficacy.

At the clinical level, some aspects also require further discussion. For instance, in this study, animals were administered an anti-PD-1 antibody intraperitoneally at 10 mg/kg every other day for 12 weeks to exacerbate HFD-induced metabolic disorders. First, while the dose is comparable to that commonly used, this treatment regimen involves a substantially higher dosing frequency than that typically used in ICI-treated mouse models (generally every 2–3 days for a total of three or four injections) ^[10–13]^ or in clinical practice (usually every 2–3 weeks) ^[14,15]^. Such frequent dosing may lead to considerable accumulation of anti-PD-1 antibodies in vivo and thereby amplify the observed metabolic effects. Additionally, this intensive dosing schedule might provoke systemic antibody-mediated responses, such as cytokine release syndrome ^[16]^, further complicating the interpretation and limiting the physiological and clinical relevance of the findings. Dose–response analyses to establish whether metabolic dysfunction occurs at clinically relevant exposure levels will need to be performed.

Second, the observation that macrophage PD-1 inhibition/deletion triggered aggravation of metabolic disorders in this study was restricted to a diet-induced metabolic disease mouse model in the absence of any tumor context. As the intricate crosstalk among cancer cells, the immune system, and metabolic organs, including adipose tissues, was not fully taken into account, the findings might not entirely capture the complex nature of ICI therapy–associated metabolic dysfunctions in cancer patients ^[17,18]^. Future studies employing syngeneic tumor models combined with anti-PD-1 treatment would more accurately recapitulate the clinical scenario. Notably, consistent with previous reports ^[19]^, the authors performed a meta-analysis of 15 randomized controlled trials and revealed that both anti-PD-1 and anti-PD-L1 therapies were related to an increased risk of systemic metabolic dysfunction in patients. Since metabolic disorders are also found in anti-PD-L1 treatment, where macrophage PD-1 is intact and functional, these clinical data cannot be fully explained by the macrophage PD-1-centered mechanism proposed here, suggesting that other unidentified mechanisms are probably involved. In other words, PD-L1 on other immune populations (such as macrophages, dendritic cells, and B cells) or non-immune cells may activate distinct pathways, and compensatory mechanisms involving other checkpoints may contribute to these metabolic effects and warrant further investigation. Of note, macrophages are the predominant immune cell type expressing PD-L1 in the tumor microenvironment ^[20]^, and PD-L1-negative macrophages have been associated with high-grade ICI therapy-related adverse events ^[21]^. Therefore, whether macrophage PD-L1 may also contribute to metabolic dysfunction following ICI treatment remains an important question for future studies.

Third, the study reports that anti-PD-1 therapy targets macrophage PD-1, thereby reducing energy expenditure and, consequently, increasing HFD-induced obesity and systemic metabolic dysfunction in mice. However, these effects were not observed in non-obese mice fed a normal diet. It is well established that obesity enhances the risk of tumor initiation and progression in patients but can paradoxically promote responses to ICI therapy, and this phenomenon is known as the “obesity paradox” in immunotherapy ^[10]^. This study adds an additional layer to this paradox by raising an interesting point: despite having enhanced therapeutic responsiveness, obese patients may also be more susceptible to ICI-induced metabolic dysfunctions.

In conclusion, the finding by Wu et al highlights a new role of PD-1 as an essential node linking immune regulation and metabolic tissue homeostasis under both physiological and pathological conditions. Although additional work is required, the identification of the mTOR–ULK1–PD-1–IRE1α axis offers druggable targets and potential predictive biomarkers for mitigating metabolic adverse events during ICI therapy without compromising anti-tumor efficacy.

## Conflict of interest

The authors declare that they have no conflicts of interest.

## Funding

C.F. is funded by the Alexander von Humboldt Professorship and by the Deutsche Forschungsgemeinschaft (German Research Foundation) under Germany’s Excellence Strategy (EXC 2030-390661388). C.F. was supported by a Cancer Research UK Programme Foundation Award (C51061/A27453).

## References

[1] Sharma P Goswami S Raychaudhuri D. Immune checkpoint therapy-current perspectives and future directions. Cell. 2023;186(8):1652-69. 37059068 10.1016/j.cell.2023.03.006

[2] Bagchi S Yuan R Engleman EG. Immune checkpoint inhibitors for the treatment of cancer: clinical impact and mechanisms of response and resistance. Annu Rev Pathol. 2021;16:223-49. 33197221 10.1146/annurev-pathol-042020-042741

[3] Chen L Han X. Anti-PD-1/PD-L1 therapy of human cancer: past, present, and future. J Clin Invest. 2015;125(9):3384-91. 26325035 10.1172/JCI80011 PMC4588282

[4] Javed SA Najmi A Ahsan W. Targeting PD-1/PD-L-1 immune checkpoint inhibition for cancer immunotherapy: success and challenges. Front Immunol. 2024;15:1383456. 38660299 10.3389/fimmu.2024.1383456 PMC11039846

[5] Topalian SL Hodi FS Brahmer JR. Safety, activity, and immune correlates of anti-PD-1 antibody in cancer. N Engl J Med. 2012;366(26):2443-54. 22658127 10.1056/NEJMoa1200690 PMC3544539

[6] Jayathilaka B Mian F Franchini F. Correction: cancer and treatment specific incidence rates of immune-related adverse events induced by immune checkpoint inhibitors: a systematic review. Br J Cancer. 2025;132(1):137. 39658607 10.1038/s41416-024-02920-3 PMC11723905

[7] Zhou L Yang S Li Y. A comprehensive review of immune checkpoint inhibitor-related diabetes mellitus: incidence, clinical features, management, and prognosis. Front Immunol. 2024;15:1448728. 39559363 10.3389/fimmu.2024.1448728 PMC11570264

[8] Wu MM Yang YC Hu ZQ. Macrophage PD-1 regulates energy expenditure and metabolic dysfunction under immune checkpoint blockade. Cell Metab. 2026;38(1):208-27.e12. 41380676 10.1016/j.cmet.2025.11.009

[9] Wu L Tsang V Clifton-Bligh R. Hyperglycemia in patients treated with immune checkpoint inhibitors: key clinical challenges and multidisciplinary consensus recommendations. J ImmunoTher Cancer. 2025;13(6):e011271. 40484647 10.1136/jitc-2024-011271 PMC12161310

[10] Bader JE Wolf MM Lupica-Tondo GL. Obesity induces PD-1 on macrophages to suppress anti-tumour immunity. Nature. 2024;630(8018):968-75. 38867043 10.1038/s41586-024-07529-3 PMC11456854

[11] Imbert C Montfort A Fraisse M. Resistance of melanoma to immune checkpoint inhibitors is overcome by targeting the sphingosine kinase-1. Nat Commun. 2020;11(1):437. 31974367 10.1038/s41467-019-14218-7 PMC6978345

[12] Lyu A Fan Z Clark M. Evolution of myeloid-mediated immunotherapy resistance in prostate cancer. Nature. 2025;637(8048):1207-17. 39633050 10.1038/s41586-024-08290-3 PMC11779626

[13] Dubrot J Lane-Reticker SK Kessler EA. In vivo screens using a selective CRISPR antigen removal lentiviral vector system reveal immune dependencies in renal cell carcinoma. Immunity. 2021;54(3):571-85.e6. 33497609 10.1016/j.immuni.2021.01.001

[14] Sehgal K Costa DB Rangachari D. Extended-interval dosing strategy of immune checkpoint inhibitors in lung cancer: will it outlast the COVID-19 pandemic? Front Oncol. 2020;10:1193. 32714874 10.3389/fonc.2020.01193 PMC7344199

[15] Jiang M Hu Y Lin G. Dosing regimens of immune checkpoint inhibitors: attempts at lower dose, less frequency, shorter course. Front Oncol. 2022;12:906251. 35795044 10.3389/fonc.2022.906251 PMC9251517

[16] Rajagopal D MacLeod E Corogeanu D. Immune-related adverse events of antibody-based biological medicines in cancer therapy. J Cell Mol Med. 2024;28(13):e18470. 38963257 10.1111/jcmm.18470 PMC11223167

[17] Lengyel E Makowski L DiGiovanni J. Cancer as a matter of fat: the crosstalk between adipose tissue and tumors. Trends Cancer. 2018;4(5):374-84. 29709261 10.1016/j.trecan.2018.03.004 PMC5932630

[18] Garner H de Visser KE. Immune crosstalk in cancer progression and metastatic spread: a complex conversation. Nat Rev Immunol. 2020;20(8):483-97. 32024984 10.1038/s41577-019-0271-z

[19] Lin MH Ye RH Jhou HJ. Immune checkpoint inhibitor therapy and risk of type 1 diabetes mellitus in metastatic cancer patients. Diabetol Metab Syndr. 2025;17(1):377. 41044787 10.1186/s13098-025-01940-0 PMC12495861

[20] Liu Y Zugazagoitia J Ahmed FS. Immune Cell PD-L1 colocalizes with macrophages and is associated with outcome in PD-1 pathway blockade therapy. Clin Cancer Res. 2020;26(4):970-7. 31615933 10.1158/1078-0432.CCR-19-1040 PMC7024671

[21] Zhou XMM Williams K Will E. PD-L1-negative macrophages are associated with poor outcomes and high-grade immune-related adverse event development in patients with melanoma receiving anti-PD-1 immunotherapy. J Invest Dermatol. 2026;146(4):1143-6. 41109454 10.1016/j.jid.2025.09.379 PMC12782841

